# Characterization of the complete chloroplast genome of orchid family species *Cymbidium bicolor* (Orchidaceae)

**DOI:** 10.1080/23802359.2019.1703591

**Published:** 2019-12-18

**Authors:** Guojia Hu, Huijuan Zhou, Shuonxin Zhang, Peng Zhao

**Affiliations:** a Key Laboratory of Resource Biology and Biotechnology in Western China, Ministry of Education, College of Life Sciences, Northwest University, Xi’an, China;; b College of Forestry, Northwest A&F University, Yangling, China

**Keywords:** *Cymbidium bicolor*, complete chloroplast genome, Illumina sequencing

## Abstract

*Cymbidium bicolor* belongs to Orchid family (Orchidaceae), it has high ornamental and traditional medicinal value. The complete chloroplast genome of *C. bicolor* was sequenced using the Illumina Hiseq platform. The size of the *C. bicolor* chloroplast genome is 156,528 bp, with an average GC content of 36.8%. This chloroplast genome has containing a large single copy (LSC) region of 85,907 bp, a small single copy (SSC) region of 17,215 bp, and two inverted (IRa and IRb) repeat regions of two 26,703 bp. A total of 124 genes were annotated, including 78 protein-coding genes, 38 tRNA genes, and 8 rRNA genes. A maximum likelihood phylogenetic tree indicated that *C. bicolor* is closely related to *C. mannii* in the genus *Cymbidium* based on 16 whole chloroplast genome sequences.

*Cymbidium bicolor* is belong to Orchid family (Orchidaceae), is a perennial herb, it has high ornamental and traditional medicinal value. It distributed in Guangdong, Hainan, Guangxi, Guizhou, and southwest Yunnan provinces in China, and this orchid plant species is also distributed in countries such as Nepal, Bhutan and India. The genus *Cymbidium* including about 55 species in tropical and subtropical Asia, south to Papua New Guinea and Australia, with 50 species distributed in China (Zhang et al. 2002). Recently, it has been published few *Cymbidium* species whole chloroplast genomes (Jiang et al. 2019). Here, we first reported complete chloroplast genomes of *C. bicolor* based on Illumina pair-end sequencing data.

The voucher specimen of *C. mannii* is stored at the herbarium of Northwest University (108°55′E, 34°15′N, accession no. SK2017200). Total genomic DNA was extracted from leaf tissue using a plant genomic DNA kit (Tiangen Biotech, Beijing, China). The whole-genome sequencing was conducted with 350 bp pair-end reads on the Illumina Hiseq platform (Illumina, San Diego, CA). After trimming, the high-quality paired-end reads were assembled with the program MITObim v1.7 (Hahn et al. 2013) using the *C. mannii* chloroplast genome sequence as a reference (GenBank accession no. NC021433) (Wang et al. 2017). Annotations were performed using the online program Dual Organellar Genome Annotator (DOGMA) (Wyman et al. 2004). The *C. bicolor* chloroplast genome sequence was submitted to GenBank (accession no. MN654912).

The chloroplast genome of *C. bicolor* was 156,528 bp in length and contains a pair of inverted repeats (IRa and IRb) regions of 26,703 bp, the large single-copy (LSC) region and small single-copy (SSC) region of 85,907 and 17,215 bp. A total of 124 genes were successfully annotated containing 78 protein-coding genes, 38 transfer RNA genes, and 8 ribosomal RNA genes.

To infer the phylogenetic position of *C. bicolor*, we constructed the phylogenetic tree based on the 16 complete chloroplast genome sequences obtained from NCBI, including six *Cymbidium* species, eight Orchid species, and two Liliaceae species as outgroups. All of the chloroplast genome sequences were aligned using MAFFT v7.0.0 (Katoh and Standley 2013). The maximum-likelihood (ML) phylogenetic tree conducted using software RAxML (Stamatakis 2006). The phylogenetic tree indicated that *C. bicolor* is closely related to *C. mannii* with 100 bootstrap support value (Figure 1). This complete chloroplast genome sequence provides the basis for future research on the evolution and molecular biology of the genus *Cymbidium* and Orchidaceae species.

**Figure 1.**
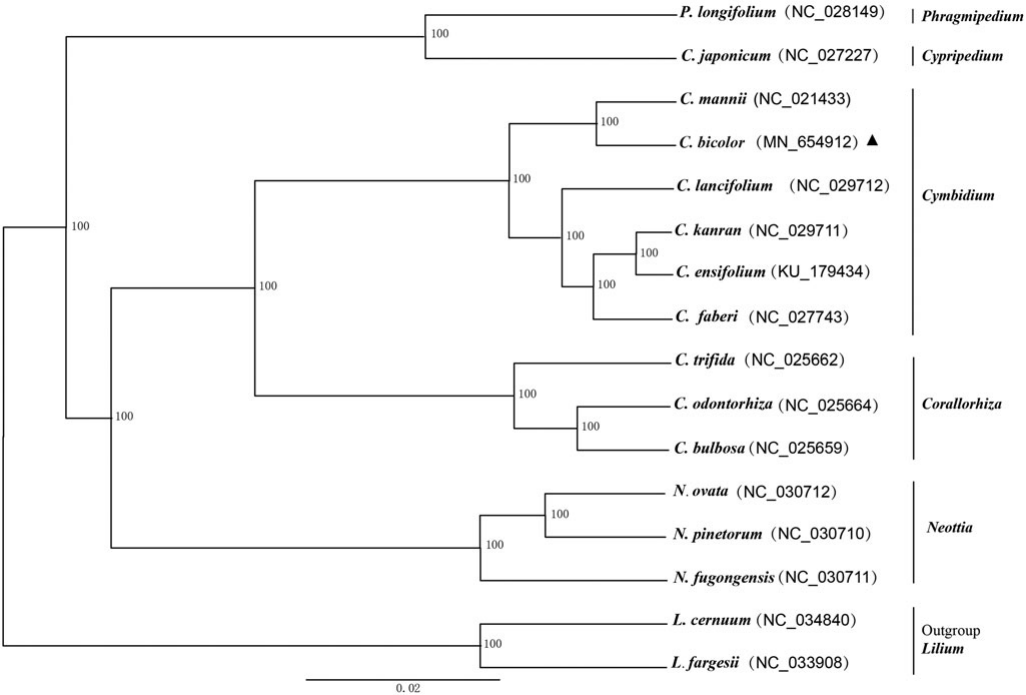
Maximum-likelihood (ML) phylogenetic tree based on 16 complete chloroplast genome sequences. The accession numbers are shown in the figure, and the triangle indicates the species *C. bicolor* sequenced in this study.
